# Stigma and Associated Sex Disparities Among Patients with Tuberculosis in Uganda: A Cross-Sectional Study

**DOI:** 10.21203/rs.3.rs-3794900/v1

**Published:** 2024-01-05

**Authors:** Juliet N. Sekandi, Trang Quach, Ronald Olum, Damalie Nakkonde, Leila Farist, Rochelle Obiekwe, Sarah Zalwango, Esther Buregyeya

**Affiliations:** Department of Epidemiology and Biostatistics, College of Public Health, University of Georgia, Athens, Georgia, USA.; Global Health Institute, College of Public Health, University of Georgia, Athens, Georgia, USA.; Makerere University School of Public Health, Kampala, Uganda.; Makerere University School of Public Health, Kampala, Uganda.; Department of Epidemiology and Biostatistics, College of Public Health, University of Georgia, Athens, Georgia, USA.; Global Health Institute, College of Public Health, University of Georgia, Athens, Georgia, USA.; Kampala Capital City Authority, Department of Public Health Service and Environment, Kampala, Uganda.; Makerere University School of Public Health, Kampala, Uganda.

**Keywords:** Self-Stigma, Anticipated-Stigma, Public-Stigma, Tuberculosis Disease, Uganda

## Abstract

**Background:**

Tuberculosis (TB) is one of the leading causes of death from a single infectious agent globally. Stigma associated with TB encompassing self-, anticipated-, and public-stigma has significant negative effects on treatment adherence. In Uganda, limited data exist on the prevalence of stigma and its relationship with sex among patients with TB. We evaluate prevalence of three types of stigma and their relationship with the sex of patients undergoing TB treatment.

**Methods:**

This cross-sectional study was conducted between July 2020 to March 2021 at selected TB clinics in Kampala, Uganda. Eligible participants were aged 18–65 with confirmed TB and starting their prescribed treatment. We collected data on socio-demographics and used 13 items to capture the self-, anticipated-, and public-stigma from which we composed the dependent variables. The primary independent variable was sex. We employed multivariable logistic regression analysis to evaluate the association between sex and the three stigma types. Additionally, we considered potential confounders such as age, HIV, and employment status. Statistical significance was defined as p<0.05.

**Results:**

In this study we enrolled 144 participants with a mean age of 35.8 years (standard deviation = 12). Half of the participants (50%, n=72) were female, 44% had a secondary education, 37.5% were unemployed, and 32.6% were co-infected with HIV. The prevalence of self-stigma was 71.1%, anticipated stigma was 75.7%, and public stigma was 41.7%. Significant factors were associated with self-stigma were female sex (adjusted odds ratio (AOR): 2.35 95% CI: 1.02–5.74) and unemployment (AOR: 2.95 95% CI: 1.16–8.58). HIV-positive status was significantly associated with anticipated stigma (AOR: 3.58 95% CI: 1.38–11.23). However, none of the variables we evaluated showed a significant association with public stigma.

**Conclusions:**

Our study showed a high prevalence of self, anticipated and public stigma among TB patients. Notably, females and unemployed individuals were at a higher risk of self-stigma, while those with HIV/AIDS and TB were more likely to report anticipated stigma. To combat stigma effectively, interventions should be tailored to cater to sex-specific needs and persons living with HIV. Future research should delve further in determinants of TB-related stigma in high-burden settings.

## INTRODUCTION

Tuberculosis (TB) remains a major public health problem and ranks second as the leading cause of death by a single infectious disease globally after COVID-19 [1]. In 2021 alone, an estimated 10.6 million new cases and 1.6 million deaths were attributed to TB globally [2]. More than two-thirds of the cases (68%) and deaths (82%) of TB-related deaths were reported in South-East Asia and the African Region alone [2]. Uganda is among the 30 countries designated by the World Health Organization (WHO) to be a high-burden country for TB/HIV co-infection, with an incidence rate of 200 cases per 100,000 and a mortality rate of 35 per 100,000 for TB [3, 4].

The WHO End TB Strategy set ambitious goals to reduce TB deaths and incidence by 95% and 90%, respectively, and to reduce to zero, the percentage of families affected by TB-related catastrophic costs by 2035, compared to 2015 [5]. Achieving these goals requires expanding patient-centered interventions, fostering cross-sectoral collaborations and commitments, and financing research and innovations while upholding equity [6]. Despite the progress over the past decades, there was an increase in TB cases and deaths in 2021 mainly attributed to disruptions in TB services caused by the COVID-19 pandemic [2, 7]. Moreover, psychosocial barriers, particularly TB-related stigma and sex disparities further complicates the realization of the End TB Strategy among others [5].

Stigma related to TB is a well-known phenomenon affecting not only the TB care continuum but also contact tracing and surveillance [8–14]. Health-related stigma is a “social process or related personal experience characterized by exclusion, rejection, blame, or devaluation that results from experience or reasonable anticipation of an adverse social judgment about a person or group identified with a particular health problem” such as TB [15]. Stigma is considered a multidimensional phenomenon due to the many institutional and societal attitudes that shape it [11]. Current literature identifies at least three types of stigma that can manifest in people living with TB [16]. *Self-stigma* is the idea that individuals may endorse negative stereotypes and behave or think according to these fake portrayals and negative messages. *Anticipated-stigma* is the worry that one will be devalued post-disclosure of a TB diagnosis. *Public-stigma* describes negative attitudes, beliefs, and behaviors held by the wider community or general public [16, 17].

Stigma is a known social determinant of health, and its presence in any form among patients with TB can result in a delay in seeking care, diagnosis and non-adherence to treatment [11]. Several studies have reported on the prevalence of stigma among patients with TB ranging from 20–80% within varying burden of disease, socioeconomic status and cultural context; however, they did not distinguish among the different types of stigma [18]. A recent study conducted in four lower-level urban clinics in Kampala, Uganda’s capital city, found the prevalence of stigma to 52% among patients with TB [19] but there was no further information about the different types of stigma. Yet, the different types of stigma could inform the design of more specific interventions.

The relationship between TB-related stigma and sex is not well-understood because results from previous studies are mixed. A study in Tanzania also suggested that men and women experience TB-related stigma differently [20]. Some studies show that females with TB may experience stigma at disproportionately higher levels than males [10, 11, 13, 21]. In contrast, a study in South Africa found that males presenting for HIV testing were more likely to have TB-related stigma than females [22]. A recent study done in Uganda did not find significant sex differences in stigma among patients with TB [19]. This study sought to estimate the prevalence of self-, anticipated-, and public-stigma and determined whether sex was associated with each type of stigma among patients with TB in Uganda.

## METHODS

### Study Design, Population and Setting

The participants were enrolled as part of a randomized controlled trial that evaluated a video observed therapy for monitoring treatment adherence among patients with TB between July 2020 to March 2021. The participants were selected treatment clinics in five health facilities that included Lubaga and Mulago hospitals, and, Kitebi, Kawaala and Kisenyi Health Center IV in Kampala. The detailed trial protocol is published elsewhere [23]. For this current study, we analyzed the cross-sectional baseline data on experiences of stigma after diagnosis of TB and initiation of treatment.

### Selection Criteria

Adults aged 18–65 with a confirmed diagnosis of drug-susceptible TB, either as a new or retreatment category, treated for no more than one month, and had provided informed consent were included. In addition, they had to be residents of Kampala for the 6-month treatment period for easy follow-up, speaking either Luganda or English. Participants were excluded if they were known to have any form of drug-resistant TB, too ill to withstand the duration of the study procedures at enrollment, had self-reported cognitive, motor, visual, or hearing disability that could hinder the proper use the assigned intervention.

### Data Collection

Data were collected using a semi-structured interviewer-administered questionnaire developed from a literature review of previous studies, translated to Luganda, and back translated to English. The interview was conducted by a trained research assistant. The baseline questionnaire collected information regarding the participants’ TB diagnosis, sociodemographic data, phone ownership, experience with smartphones and technology, transportation, social and family support, privacy concerns, personal knowledge of TB, and community perception of TB [23].

### Key Variables and Definitions

Sex, measured as male or female, was the primary independent variable of interest. The dependent variables were self-stigma, anticipated stigma, and public stigma. Other variables included age, level of education, religion, marital status, number of other household members, and HIV status at baseline.

### Measurement of Stigma

A total of 13 items adopted from USAID’s TB Stigma Measurement Guidance were used to construct composite variables of the types of stigmas [17]. Self-stigma was measured using three question items; anticipated-stigma was constructed from three items, and public-stigma from seven items. First, the response questions about stigma originally captured with a 4-point Likert scale (1=’agree’, 2=’strongly agree’, 3=’disagree’, and 4=’strongly disagree’) were converted to dichotomous responses. The responses ‘agree’ and ‘strongly agree’ were then collapsed to ‘Yes,’ while ‘disagree’ and ‘strongly disagree’ were collapsed to ‘No.’ A composite variable of stigma was created based on responses to the specific domain question items to determine the presence or absence of the three types of stigmas. For example, if the response was yes on any of the three questions assigned to self-stigma, the outcome was coded “1 = Yes”; if none, it was coded as “0 = No”. The same data processing was repeated to create a composite outcome variable for anticipated- and public- stigma.

### Data Analyses

Descriptive statistics, including frequencies, percentages, mean, standard deviation (SD), median and interquartile range (IQR) were done. The overall prevalence for each type of stigma was estimated and then stratified by sex. To compare the distribution of the types of stigma across sex, we conducted chi-square tests. Univariate and multivariable logistic regression analyses were performed to evaluate the associations between sex and self-, anticipated- and public- stigma. Age, education, employment status, number of household members, and HIV status were covariates considered potential confounders of the sex and stigma relationship. Crude and adjusted Odds Ratios (OR) were presented with a 95% Confidence Interval (CI) and P-values. Associations with p < 0.05 were considered significant. All statistical analyses were conducted using SAS version 9.4 (SAS Institute, Cary, NC).

## RESULTS

A total of 144 patients with TB, half of whom (50%) were females, were enrolled. The mean age was 35.8 years (SD = 12.0), and about one-third between the ages of 24 and 34 (Table 1). Nearly half (44.4%) of participants had secondary school as the highest level of education, 45% were married, and 37.5% were unemployed. The mean number of other household members was 4.3 (SD = 2.7), with more than half (56.9%) reporting one to four other household members. Over one-third of the participants (31.9%) self-reported as HIV-positive at baseline.

### Item Analysis of Stigma Questions Stratified by Sex

Of the three items assessing self-stigma, the vast majority (70.1%) of participants reported feeling uncomfortable taking their TB medicine in the presence of any person from their community. Moreover, females (75% vs. 25%) expressed discomfort in taking their medication publicly than males. Additionally, 70.1% of the participants reported feeling anticipated stigma expressed as the feeling that people in their community would not offer support if they were aware of their TB diagnosis. More women than men (76.4% vs 63.9%) anticipated getting no community social support than men. Overall public-stigma was low, ranging from 8.3–27.1% as expressed through the responses to the seven items that were asked in relation to TB (Table 2).

### Prevalence of Stigma, Sex Differences and Associated Factors

#### Self-Stigma

**Figure 1:**
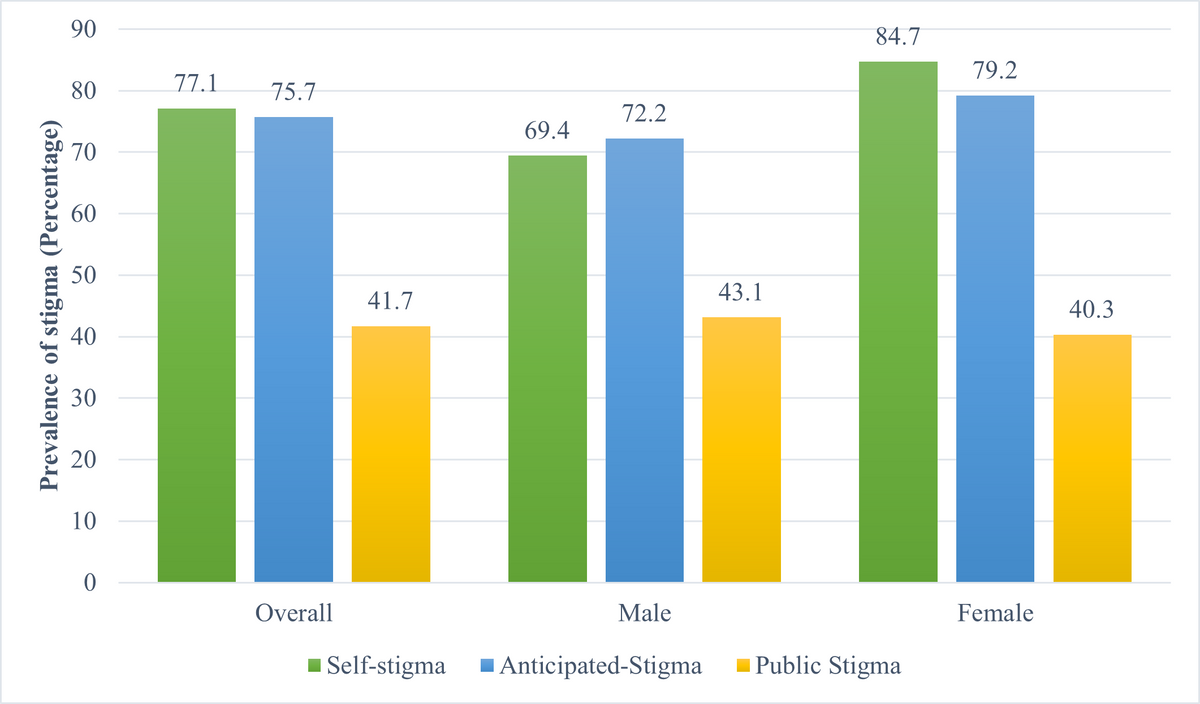
Prevalence of the Three Types of Stigma Stratified by Sex.

The overall prevalence of self-stigma was 77.1% and there was a significant sex difference with females having a higher level than males (87.4% vs 69.4%, p = 0.047, Fig. 1). This association was established in multivariate logistics regression. Simple logistic regression analysis indicated that being female was significantly associated with reported self-stigma in patients with TB (crude odds ratio (COR): 2.44, 95% CI: 1.10–5.68, Table 3). After adjusting for covariates, female patients with TB were 2.35 times more likely to report self-stigma (95% CI 1.02–5.74) compared to their male counterparts. Unemployed patients with TB were also 2.95 times more likely to report self-stigma (95% CI: 1.16–8.58) than employed patients.

#### Anticipated Stigma

Anticipated stigma was present in 75.7% of patients with TV but this was not significantly different by sex (79.2% vs. 72.2%, p = 0.437, Fig. 1). At multivariable logistic regression, female patients with TB were more likely to report anticipated stigma but this association was not statistically significant after adjusting for confounders (AOR: 1.64, 95% CI: 0.74–3.71). Being HIV positive was significantly associated with anticipated stigma (AOR: 3.58, 95% CI: 1.38–11.23) after adjusting for confounding by sex and employment (Table 4).

#### Public Stigma

Finally, less than half (41.7%) of the patients had public stigma, with slightly fewer women having public stigma than men. This was however not statistically significant (40.3% vs. 41.7%, p = 0.866, Fig. 1). Sex or other factors evaluated were not associated with public stigma at simple and multivariable logistic regression analyses (Table 5).

## Discussion

In this study we aimed to estimate the prevalence of three types of stigma related to TB and their association with sex among patients confirmed to have disease in Kampala, Uganda. We found a high prevalence of self-stigma and anticipated-stigma and low level of public-stigma. We also found that being female and HIV infected were significantly associated with self- and anticipated stigma respectively. To our knowledge, this study is among the first to examine the three types of stigma among patients with TB in Uganda.

Self-stigma refers to the concept where individuals internalize negative stereotypes and adjust their behaviors or thoughts based on these inaccurate and negative representations [16]. Our study found a higher prevalence of three types of stigma in patients with TB compared to other published studies. Two studies in Zambia and Ethiopia specifically examined self-stigma among TB patients and reported a prevalence of 48.3% and 50.4%, respectively [13, 24]. These levels are lower than the prevalence of 77.1% for self-stigma reported in our study. The differences in prevalence between our study and other studies could be due to variations in stigma measurement, sample size, cultural context, and settings. The study in Zambia utilized three questions adopted from the literature to assess stigma, whereas the Ethiopian study adopted items from a generic guide by the WHO [13, 24]. Our results align with the 2020–2024 Uganda National Strategic Plan that highlights the need for targeted interventions to reduce self-stigma among people living with TB [25].

Anticipated-stigma also known as perceived-stigma refer to the fear that revealing a TB diagnosis will lead to being perceived less favorably by others [16]. We found that nearly three in four patients (75.7%) with tuberculosis in Uganda experience anticipated stigma. This prevalence is higher than levels of anticipated/perceived stigma in patients with TB that ranged from 42.4% to 52% reported in other studies done in Uganda, Ethiopia, India and Cambodia [19, 26–28]. In India, the prevalence of perceived stigma among patients with TB when dealing with family and friends was 45.5% and 58.2% when at the workplace [27]. These studies were conducted in variable settings including both urban and rural TB clinics, and community settings. They used different scales than our study to measure stigma. Some used the Van Rie Scale [29] while others used new or adopted scales to measure anticipated-stigma.

Finally, public-stigma refers to the collective negative perceptions, beliefs, and actions exhibited by the broader society or general populace towards specific groups or issues [16]. In this study, we reported a prevalence of public stigma of 41.7%. It also corroborates findings among Uganda’s general population, which found that 47% had stigmatizing attitudes towards TB [30]. However, a study done in India reported a much higher level of 71.6% for public/social stigma [31]. The differences observed could be partly due to the differences in socioeconomic, cultural, and living conditions in the different countries and settings, in addition to the heterogeneity in the measurement of stigma. In the Indian study, public stigma was conducted within the community using house-to-house surveys, and telephone interviews. The study also utilized the 8-item stigma scale for chronic illnesses (SSCI) to assess public stigma, which could potentially explain the difference with our study [31].

Most studies thus far have reported on general TB-related stigma on a numerical scale without distinguishing the specific type of stigma. In South Africa, individuals with presumptive TB had a higher stigma score than those already diagnosed with TB [32]. In Ethiopia, patients with TB had a higher stigma score compared to their families and the general population [24]. Among Kenyan pastoralists diagnosed with TB, the mean scores for experienced stigma were higher than perceived/anticipated stigma [21]. Other studies in China [33, 34] and Vietnam [35] have also reported higher stigma scores among patients with TB. A higher mean stigma score has also been reported among patients with multi-drug-resistant TB in South Africa [36]. It is important to note that most of the studies used different tools to assess stigma, making it difficult to compare findings across studies, within and between settings. This challenge underscores the need for the methods proposed in the TB stigma measurement guidance [37].

Our study showed that females were over two times more likely to report self-stigma than males and this association was statistically significant. Similar findings have also been reported in Zambia and China [13, 33]. In Zambia, the study found that female patients with TB were 5.47 times more likely to experience overall stigma than male patients [13]. However, two studies conducted in China found that TB stigma was not associated with sex [34, 38]. In two studies in Uganda [19] and South Africa [32], no sex differences in experiences of TB-related stigma were reported. These variations could be due to the differences in the scale used to measure stigma, sample sizes, setting and related sociocultural factors. In our study, females were more likely to anticipate being without social support than men. Women tend to internalize feelings of stigma more than men due to underlying cultural norms and gender-specific norms [39]. Unmarried females are more likely to express feelings of shame and worsened self-esteem resulting from fear that having TB will ruin their marriage prospects [20, 40]. In addition, feelings of self-stigma often occur among married females, who worry about receiving potential rejections from their spouses as well as the inability to continue their domestic role as household's primary caregiver if they have TB [13, 41].

A large systematic review suggested that the higher prevalence of TB-related stigma is associated with sex differences in financial independence in many low-income countries [18]. Women often depend on men for financial support, including for obtaining TB care and treatment. In some settings, gender inequity and harmful cultural practices are still prevalent with underlying social consequence for women who suffer from TB [12, 18, 20]. Although our study did not find an association between female sex and public stigma, other studies in Bangladesh and Zambia found female sex to be a significant predictor of anticipated and public stigma in patients with TB [10, 13]. More research is needed to understand the mechanisms under which gender modulates stigma experiences among patients with TB to guide the design of interventions.

Unemployed patients with TB were almost three times more likely to report self-stigma than those who were employed. Our findings are consistent with a multi-country study conducted in Bangladesh, India, Malawi, and Colombia found that in Malawi, unemployment was more likely to be linked to a greater prevalence of stigma in females than in males [34]. The inability to work due to frequent clinic visits is associated with higher reporting of stigma[42]. Fear of job loss and reduced family income is often reported among individuals with TB [24]. One possible explanation for this observed relationship is the perception of productivity and societal contribution. In many cultures, employment is seen as a marker of societal value and self-worth [43].Consequently, unemployed individuals may already feel marginalized or stigmatized, which may be exacerbated upon receiving a TB diagnosis. In addition, unemployment further worsens the impact of catastrophic spending among patients with TB and their families. More than half (53.1%) of Ugandan households experience catastrophic TB-related costs, primarily due to non-medical costs like transportation, dietary supplements, and food [44].

In our study, persons living with HIV were over 3.5 times more likely to report anticipated- stigma than patients without HIV infection. This is a key finding for Uganda since it is among the 30 high-burden countries for TB/HIV co-infection [2]. Anticipated stigma in the form of fear of disclosure is prevalent among people living with HIV [45]. In our study, one-third of participants self-reported having HIV coinfection and such the high level of anticipated-stigma may not be surprising [4]. Our finding is consistent with several studies among people living with HIV and tuberculosis. Two studies conducted in Ethiopia found HIV status linked to increased odds of reporting perceived stigma among patients with TB [26, 46]. Additional studies indicate similar findings [47] as well as a relationship between HIV status and overall stigma in patients with TB[48]. Syndemic stigma is likely underlying what we observed in our study, where two simultaneously occurring HIV and TB epidemics with their related stigmas are intertwined [37]. The linkage between the two diseases may often cause compound effects of stigma, making it difficult to distinguish between them [11]. It is important to target special efforts to support people who are TB/HIV co-infected, as they are likely to mitigate the negative impacts of stigma.

Moreover, individuals co-infected with TB/HIV and carrying feelings of anticipated stigma can possibly transmit the infection to others while remaining fearful of disclosing their diagnosis [41]. This is further exacerbated by misinformation within communities that often associate TB infection with HIV [19]. Community-based interventions should be of focus when setting sights on reducing anticipated stigma among TB/HIV coinfected individuals. Mass media campaigns that disseminate information about TB and HIV that simultaneously dispels myths can help normalize having either diagnosis [49]. In addition, integrating tuberculosis care with less-stigmatizing health conditions such as diabetes and hypertension instead of HIV/AIDS could help reduce TB-related stigma for persons not living with HIV [9]. A larger study in the future should be carried out to further assess other factors associated with each form of stigma, including public stigma, and the impact of each type of stigma on treatment adherence. The role of digital interventions in addressing such barriers should also be explored [23].

Our study has several implications for TB care and public health policy in Uganda and potentially other similar settings. It reveals a higher prevalence of self-stigma and anticipated stigma among TB patients in Kampala, surpassing levels reported in other African countries and other settings. The increased vulnerability of females and unemployed patients to self-stigma and those with TB/HIV coinfection to anticipated stigma calls for tailored interventions that specifically address these susceptibilities[50]. Emphasizing gender-sensitive approaches, integrating socioeconomic support systems, and recognizing the compounded challenges of dual diagnoses become paramount. Additionally, community-based initiatives, proactive use of mass media for dispelling myths and misinformation, embracing digital health technologies, and integrating TB care with other health conditions can serve as essential strategies in mitigating stigma. Addressing these multifaceted issues is crucial for enhancing early diagnosis, improving treatment adherence, and fortifying overall TB prevention efforts in Uganda.

### Strengths and Limitations

Our study is among the first in the Ugandan setting to examine the relationship between sex and the various types of stigma among patients with TB. However, it is not without limitations. First, we used a cross-sectional design which provides a snapshot of the outcome. We cannot make causal inferences about type of stigma since it can vary over time. There were some noticeable magnitude and positive direction of effect despite not being statistically significant which could have been due to a limited sample size. The study participants were only drawn from public urban clinics; this limits the generalizability of our findings to private clinics or rural settings in Uganda.

## CONCLUSIONS

Our study showed a high prevalence of self, anticipated and public stigma among TB patients. Notably, females and unemployed individuals were at a higher risk of self-stigma, while those with HIV/AIDS and TB were more likely to report anticipated stigma. To combat stigma effectively, interventions should be tailored to cater to sex-specific needs and persons living with HIV. Future research should delve further into determinants of TB-related stigma in high-burden settings.

## Figures and Tables

**Table 1:** Baseline Characteristics of Patients with TB Enrolled in the Study

Variables (N = 144)	Response	Frequency	Percentage
Age in years	Mean (SD)	35.8	12.0
Age Groups	18-24	27	18.8
	25–34	48	33.3
	34-44	30	20.8
	≥45	39	27.1
Sex	Female	72	50.0
	Male	72	50.0
Education	None/Primary	55	38.2
	Secondary	64	44.4
	Tertiary or University	25	17.4
Religion	Catholic	51	35.4
	Protestant	33	22.9
	Muslim	32	22.2
	Pentecostal	19	13.2
	Other	9	6.3
Marital Status	Single	66	45.8
	Married/Cohabiting	55	38.2
	Previously Married	23	16.0
Employed	Yes	90	62.5
	No	54	37.5
Average number of other Household Members	Median (IQR)	4.0	3.0
	0	2	1.4
	1–4	82	56.9
	5–13	60	41.7
HIV Status	Negative	98	68.1
	Positive	46	31.9

**Table 2:** Responses to 13-item stigma questions stratified by sex

Variables	Total N (%)	Male (n = 72)	Female (n = 72)	p-value
**Self-stigma**				
Do you feel afraid or ashamed of telling any family or household members about your TB diagnosis				
No	111 (77.1)	60 (83.3)	51 (70.8)	0.113
Yes	33 (22.9)	12 (16.7)	21 (29.2)	
How would you feel if anyone in the community found out about your Tb diagnosis				
Not worried	83 (57.6)	47 (65.3)	36 (50.0)	0.092
Shame/Worry	61 (42.4)	25 (34.7)	36 (50.0)	
Would you be comfortable taking your TB medicine in the presence of any person from your community				
No	101 (70.1)	47 (65.3)	54 (75.0)	0.275
Yes	43 (29.9)	25 (34.7)	18 (25.0)	
**Anticipated stigma**				
Do you think it would be difficult for you to ask your family or household members for the support and care you need because you have TB				
No	109 (75.7)	55 (76.4)	54 (75.0)	0.980
Yes	33 (22.9)	16 (22.2)	17 (23.6)	
Don’t know	2 (1.4)	1 (1.4)	1 (1.4)	
If your family or household members find out about your TB diagnosis, how do you think it will affect your relationship with them				
No support	18 (12.5)	9 (12.5)	9 (12.5)	1.000
Support	126 (87.5)	63 (87.5)	63 (87.5)	
Do you think that people in your community would offer any needed support to you even if they know you have TB disease				
No	101 (70.1)	46 (63.9)	55 (76.4)	0.149
Yes	27 (18.8)	18 (25.0)	9 (12.5)	
Don’t know	16 (11.1)	8 (11.1)	8 (11.1)	
**Public stigma**				
Some people may not want to eat or drink with friends who have TB				
No	129 (89.6)	62 (86.1)	67 (93.1)	0.275
Yes	15 (10.4)	10 (13.9)	5 (6.9)	
Some people feel uncomfortable about being near a person who has had TB				
No	130 (90.3)	66 (91.7)	64 (88.9)	0.778
Yes	14 (9.7)	6 (8.3)	8 (11.1)	
Some people do not want those with TB playing with their children				
No	121 (84.0)	61 (84.7)	60 (83.3)	1.000
Yes	23 (16.0)	11 (15.3)	12 (16.7)	
Some people keep their distance from people with TB				
No	132 (91.7)	65 (90.3)	67 (93.1)	0.763
Yes	12 (8.3)	7 (9.7)	5 (6.9)	
Some people do not want to talk to others with TB				
No	112 (77.8)	51 (70.8)	61 (84.7)	0.071
Yes	32 (22.2)	21 (29.2)	11 (15.3)	
Some people may not want to eat or drink with family members who have TB				
No	123 (85.4)	61 (84.7)	62 (86.1)	1.000
Yes	21 (14.6)	11 (15.3)	10 (13.9)	
Prefer not to have people with TB living in their community				
No	105 (72.9)	54 (75.0)	51 (70.8)	0.708
Yes	39 (27.1)	18 (25.0)	21 (29.2)	

**Table 3:** Logistic Regression Analysis of Factors Associated with Self-stigma

Variable	No Self Stigma n (%)	Self-Stigma n (%)	Crude OR (95% CI)	Adjusted OR (95% CI)
Sex				
Male	22 (30.6)	50 (69.4)	1.00	1.00
Female	11 (15.3)	61 (84.7)	**2.44 (1.10–5.68)**	**2.35 (1.02–5.74)**
Age category (years)						
45–65	9 (23.1)	30 (76.9)	1.00	
35–44	11 (36.7)	19 (63.3)	0.52 (0.18–1.48)	
25–34	7 (14.6)	41 (85.4)	1.76 (0.59–5.43)	
18–24	6 (22.2)	21 (77.8)	1.05 (0.33–3.55)	
Highest level completed in school				
No education or primary	12 (21.8)	43 (78.2)	1.00	
Secondary	14 (21.9)	50 (78.1)	1.00 (0.41–2.39)	
Tertiary/University	7 (28.0)	18 (72.0)	0.72 (0.25–2.20)	
Marital status						
Married	13 (23.6)	42 (76.4)	1.00	
Previously married	4 (17.4)	19 (82.6)	1.47 (0.45–5.75)	
Single/never married	16 (24.2)	50 (75.8)	0.97 (0.41–2.24)	
Are you currently employed						
Yes	27 (30.0)	63 (70.0)	1.00	1.00
No	6 (11.1)	48 (88.9)	**3.43 (1.39–9.78)**	**2.95 (1.16–8.58)**
Number of other household members				
1–4	20 (24.1)	63 (75.9)	1.00	
5–13	13 (22.0)	46 (78.0)	1.12 (0.51 – 2.53)	
HIV status				
HIV negative	24 (24.7)	73 (75.3)	1.00	1.00
HIV positive	8 (17.4)	38 (82.6)	1.56 (0.66–4.01)	1.68 (0.69–4.43)

**Table 4:** Logistic Regression Analysis of Factors Associated with Anticipated Stigma

Variables	No Anticipated Stigma: n (%)	Anticipated Stigma: n (%)	Crude OR (95% CI)	Adjusted OR (95% CI)
Sex				
Male	20 (27.8)	52 (72.2)	1.00	1.00
Female	15 (20.8)	57 (79.2)	1.46 (0.68–3.19)	1.64 (0.74–3.71)
Age category (years)				
45–65	8 (20.5)	31 (79.5)	1.00	
35–44	9 (30.0)	21 (70.0)	0.60 (0.20–1.82)	
25–34	11 (22.9)	37 (77.1)	0.87 (0.30–2.41)	
18–24	7 (25.9)	20 (74.1)	0.74 (0.23–2.40)	
Highest level you completed in school				
No education or primary	10 (18.2)	45 (81.8)	1.00	
Secondary	18 (28.1)	46 (71.9)	0.57 (0.23–1.34)	
Tertiary/University	7 (28.0)	18 (72.0)	0.57 (0.19–1.79)	
Marital status				
Married	10 (18.2)	45 (81.8)	1.00	
Previously married	3 (13.0)	20 (87.0)	1.48 (0.40–7.13)	
Single/never married	22 (33.3)	44 (66.7)	0.44 (0.18–1.02)	
Are you currently employed				
Yes	24 (26.7)	66 (73.3)	1.00	
No	11 (20.4)	43 (79.6)	1.42 (0.64–3.29)	
Number of other household members				
1–4	20 (24.1)	63 (75.9)	1.00	
5–13	14 (23.7)	45 (76.3)	1.02 (0.47–2.26)	
HIV status				
HIV negative	29 (29.9)	68 (70.1)	1.00	1.00
HIV positive	5 (10.9)	41 (89.1)	**3.50 (1.35–10.91)**	**3.58 (1.38–11.23)**

**Table 5:** Logistic Regression Analysis of factors associated with public stigma.

Variables	No Public Stigma n (%)	Public Stigma n (%)	Crude OR (95% CI)
Sex			
Male	41 (56.9)	31 (43.1)	1.00
Female	43 (59.7)	29 (40.3)	0.89 (0.46–1.73)
Age category (years)			
45–65	25 (64.1)	14 (35.9)	1.00
35–44	14 (46.7)	16 (53.3)	2.04 (0.78–5.48)
25–34	29 (60.4)	19 (39.6)	1.17 (0.49–2.83)
18–24	16 (59.3)	11 (40.7)	1.23 (0.44–3.38)
Highest level you completed in school			
No education or primary	37 (67.3)	18 (32.7)	1.00
Secondary	35 (54.7)	29 (45.3)	1.70 (0.81–3.64)
Tertiary/University	12 (48.0)	13 (52.0)	2.23 (0.85–5.94)
Marital status			
Married	30 (54.5)	25 (45.5)	1.00
Previously married	16 (69.6)	7 (30.4)	0.53 (0.18–1.44)
Single/never married	38 (57.6)	28 (42.4)	0.88 (0.43–1.82)
Are you currently employed			
Yes	53 (58.9)	37 (41.1)	1.00
No	31 (57.4)	23 (42.6)	1.06 (0.53–2.10)
Number of other household members			
1–4	51 (61.4)	32 (38.6)	1.00
5–13	32 (54.2)	27 (45.8)	1.34 (0.68–2.65)
HIV status			
HIV negative	59 (60.8)	38 (39.2)	1.00
HIV positive	25 (54.3)	21 (45.7)	1.30 (0.64–2.65)

## Data Availability

Deidentified data are available upon request and agreement to terms of data sharing and use at the University of Georgia and Makerere University.
